# High-throughput sequencing-based gene profiling on multi-staged fruit development of date palm (*Phoenix dactylifera,* L.)

**DOI:** 10.1007/s11103-012-9890-5

**Published:** 2012-02-21

**Authors:** Yuxin Yin, Xiaowei Zhang, Yongjun Fang, Linlin Pan, Gaoyuan Sun, Chengqi Xin, Mohammed M. Ba Abdullah, Xiaoguang Yu, Songnian Hu, Ibrahim S. Al-Mssallem, Jun Yu

**Affiliations:** 1 Joint Center for Genomics Research (JCGR), King Abdulaziz City for Science and Technology (KACST) and Chinese Academy of Sciences (CAS), Riyadh, Kingdom of Saudi Arabia; 2 CAS Key Laboratory of Genome Sciences and Information, Beijing Institute of Genomics, Chinese Academy of Sciences, Chaoyang District, Beijing, China; 3 Graduate University of Chinese Academy of Sciences, Shijingshan District, Beijing, China; 4 Department of Biotechnology, College of Agriculture and Food Sciences, King Faisal University, Al-Hssa, Hofuf Kingdom of Saudi Arabia

**Keywords:** Date palm, Transcriptome, Fruit, Development stage

## Abstract

Date palm provides both staple food and gardening for the Middle East and North African countries for thousands of years. Its fruits have diversified significantly, such as nutritional content, size, length, weight color, and ripping process. Dates palm represent an excellent model system for the study of fruit development and diversity of fruit-bearing palm species that produce the most versatile fruit types as compared to other plant families. Using Roche/454 GS FLX instrument, we acquired 7.6 million sequence tags from seven fruiting stages (F1–F7). Over 99% of the raw reads are assembled, and the numbers of isotigs (equivalent to transcription units or unigenes) range from 30,684 to 40,378 during different fruiting stages. We annotated isotigs using BLASTX and BLASTN, and mapped 74% of the isotigs to known functional sequences or genes. Based on gene ontology categorization and pathway analysis, we have identified 10 core cell division genes, 18 ripening related genes, and 7 starch metabolic enzymes, which are involved as nutrition storage and sugar/starch metabolisms. We noticed that many metabolic pathways vary significantly during fruit development, and carbohydrate metabolism (especially sugar synthesis) is particularly prominent during fruit ripening. Transcriptomics study on various fruiting stages of date palm shows complicated metabolic activities during fruit development, ripening, synthesis and accumulation of starch enzymes and other related sugars. Most Genes are highly expressed in early stages of development, while late developmental stages are critical for fruit ripening including most of the metabolism associated ones.

**Electronic supplementary material:**

The online version of this article (doi:10.1007/s11103-012-9890-5) contains supplementary material, which is available to authorized users.

## Background

Date palm (*Phoenix dactylifera* L. 2n = 36) (Solimann and Al-Mayah 1978) is a sociocultural symbol for Arabian Peninsula (Saudi Arabia and Gulf countries) with long agriculture history (Morton 1987). And it is a major food crops in the Middle East, Northern Africa, and many other places with suitable climates (Al-Mssalllem 1996). All vegetative and reproductive parts of this plant species are of some economic, nutritional, or medicinal importance. Date palm used to be the main nutrient for the inhabitants of Arabian Peninsula. Nearly a thousand cultivars have been distinguished and over 350 cultivars are distributed in the Kingdom of Saudi Arabia, mainly in Al-Hssa Oasis. Genomic data for the date palm have just started to become available (Al-Dous et al. 2011), (Yang et al. 2010) but detailed and high-quality gene annotations and genome assembly are yet to come.

The edible parts of *P. dactylifera* is the ripen fruit and bud. It has high nutritional value and contains a variety of vitamins, fiber, sugars and trace amount of fat and proteins (Al-Shahib and Marshall 2003). The date ripening is a complex process, in which major metabolic pathways undergo gradual switches toward the terminal sugar-rich stage, and it may share many similar characteristics to those of other fruits of different plant families. In the early developmental stage, fruit tissues undertake several rounds of cell division, followed by nutrient, metabolite, and energy storing during cell expansion (Gillaspy et al. 1993; Giovannoni 2001, 2004). The frutis are subsequently ripening and undergoing a series of changes that starch converts into small molecules, such as easily absorbed compounds: monosaccharides and volatile secondary metabolites (Janssen et al. 2008). A very recent publication sequenced EST data of 5 developmental stages of oil palm (*Elaeis guineensis* Jacq) and date palm fruits, and put their emphasis on oil synthesis pathway and transcriptomics regulatory details for the oil palm(Bourgis et al. 2011).

Recently, genomic approaches have been applied to investigate fruit development (Giovannoni 2004; Aharoni et al. 2000; Trainotti et al. 2005). Other than serial analysis of gene expression (SAGE) approach (Velculescu et al. 1995) and various microarray-based methods, the next-generation sequencing platforms provide powerful tools for rapid acquisition of transcriptomes that offer better opportunities to compare gene expressions at different fruiting stages. In this study, we employed the GS FLX (Roche/454) sequencing technology (Margulies et al. 2005; Jarvie and Harkins 2008) to acquire for the first time massive transcriptomics data for the date palm fruit at seven different developmental stages from an elite local cultivar Khalas. This one-million-per-library sampling allows us to assemble the high-quality and long sequences into large contigs or isotigs (termed based on the computational definition of the specialized manufacturer-designed software). Therefore, we are able to annotate most of the isotigs and obtained meaningful analysis results by comparing gene expression profiles of the defined fruiting stages and identify genes that are relevant to biological functions and genetic traits. Our data provide the date palm research community an ample genomic information for future investigations into fruit development and pathway profiling for nutrients, paving a way for in-depth biological and molecular studies.

## Results

### Data acquisition and sequence assembly

**Fig. 1:**
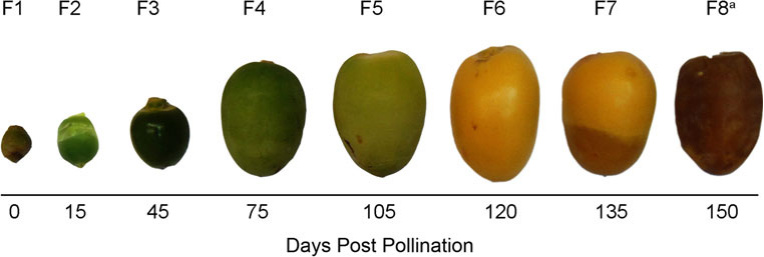
Date palm fruits (dates) at eight developmental stages. The samples of cultivar Khalas are collected at the corresponding days from Al-Hssa and Al- Kharj, Kingdom of Saudi. Days post pollination are showed at the *bottom*. a We are unable to isolate RNA from this stage

We harvested all the date samples from palm farms and defined their developmental or fruiting stages based on field observations and community norms in Al-Hssa Oasis (Fig. 1). F1 and F2 stage represent a Kimri (Jimiri) stage (1–30 days post pollination) which characterized by high cell multiplication and the fruit is hard and apple green. We have not calculated the exact time elapsed between pollination and the beginning of kimiri stage; F3 and F4 represent a Khalal stage (31–90 days post pollination), cell multiplication and expansion, starch accumulation and color development; F5 and F6 represent a Beser stage (91–120 days post pollination) cell expansion, color and taste change from green to yellow or red and not tasted to sweet respectively; F7 represent a Ratab stage (121–150) days post pollination) color change gradually from yellow or red into light to dark brown of black, accumulation of sugars; the last stage, F8, represents the Tamar stage (151–180), which is the ripened fruit of date palm and conversion of sugars into glucose and fructose. Unfortunately, from F8 stage (Tamar) and we were not able to isolate enough RNA for the transcriptomics study. Therefore, we sequenced these libraries of seven fruiting stages (F1–F7) separately, and yielding consistent data for each fruiting stage (Table 1). We acquired a total of greater than 7.6 million reads in an average length of 361 bp (with a median length of 399 bp). The longest read is 967 bp in length (Table 1). More than 58% of the sequences are in a range of 300–500 bp (Additional file 1, Figure S1 A). The GC contents of sequence reads are slightly different; F1 has a higher read coverage at 40–45% (27.15% of the stage-based data) and F7 has a higher read coverage at 60–65% (11.40% of the stage-based data) (Additional file 1, Figure S1).

**Table 1:** An overview of raw data and assemblies from Roche/454 GS FLX sequencing

Sample	Raw data			Assembly				
	Reads	Total bases	Average length (bp)	Reads in assembly	%	Isotigs	Average length (bp)	GC content
F1	1,061,314	404,794,144	381.32	1,060,821	99.95	38,727	983	45.30
F2	1,054,534	343,621,741	325.78	1,053,217	99.88	30,684	712	45.45
F3	1,026,640	381,908,118	371.92	1,026,093	99.95	31,762	855	46.16
F4	1,054,961	379,163,526	359.38	1,054,703	99.98	40,378	922	45.05
F5	1,175,621	431,874,515	367.28	1,175,284	99.97	30,287	884	45.53
F6	1,142,106	433,201,759	379.18	1,141,841	99.98	32,855	951	45.52
F7	1,111,851	382,375,060	343.81	1,111,548	99.97	38,244	759	45.80
All	7,627,027	2,756,938,863	361.39					

We assembled our raw data using the latest Newbler 2.5 software (Kumar and Blaxter 2010). We first assembled the raw reads from each library independently and then assembled all raw reads from the seven libraries together. Based on the read vs. isotig saturation curve (Additional file 2, Figure S2), it is clear that the numbers of isotigs is still increasing even though there is a slowing down turning point around 300,000 reads. For eukaryotic cells, the isotig saturation boundary is roughly 10 million reads (data not shown). Although different library seems have a slightly different curve, we believe that they are within the noise of deviation. It is very encouraging that more than 99% of the raw sequences are actually assembled into isotigs, and the numbers of isotigs are within an expected range: the highest number (40,378) in F4 and the lowest number (30,684) in F2 (Table 1). The average isotig lengths are ranging 712–983 bp, and 63.6% of the sequences length in a range of 500–2,500 bp (Additional file 1, Figure S1 C). In addition, the average GC content of all isotigs is about 45% albeit a few percent deviations (Additional file 1, Figure S1 2 D).

**Fig. 2:**
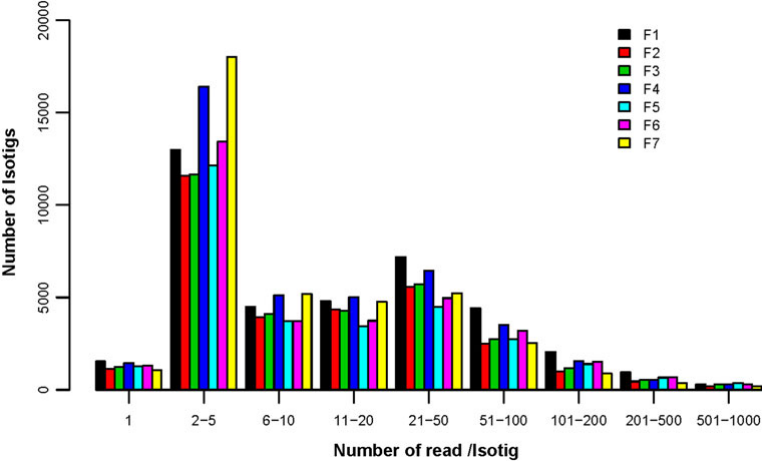
The read coverage of the assembled isotigs from all sequencing reads

The isotig coverage is overall quite satisfactory in that only 3.7% of the isotigs are contributed by single reads and ~50% of the isotigs are composed of 2–10 reads (Fig. 2). The F7 library gave rise to 60.7% multiple-read isotigs. The overall distribution is also normal; majority of the isotigs are low-coverage (2–5 reads) and very small fraction of them are high-coverage (over 100 reads). Since the sampling was not aiming for saturation, our analysis is actually focused on annotation to gain an overview for the fruiting process of the date palm.

### Isotig sequence annotations

**Fig. 3:**
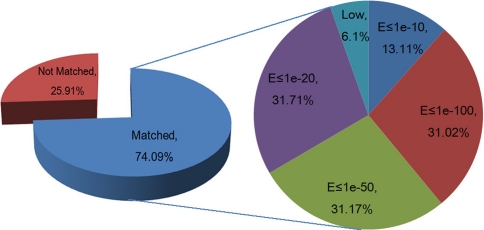
Annotated isotigs at different E-values. ‘Low’ and ‘No match’ indicate isotigs identified with an interval of 1.0 × 10^−10^ < E ≤ 1.0 × 10^−5^ and no homology detected at the same interval for any known sequences in the public databases, respectively

To annotate all isotigs, we first compared them to the NCBI non-redundant (nr) database using BlastX and then searched the unmatched isotigs against the NCBI nucleotide (nt) database using BlastN. The E value cutoffs are set as ≤1e − 5 for BlastX and ≤1e − 10 for BlastN (Table 2). After two rounds of intensive analyses, we found that ~74% of the isotigs match to known functional genes (Fig. 3). When we increased the E values to ≤1e − 100 and ≤1e − 50, there are still 20.32% and 20.42% of the isotigs being annotated, respectively? Only 4% lies between 1e − 10 and 1e − 5.

**Table 2:** Search results from public non-redundant database

Isotigs		NCBI-BLAST		No-hits^b^
		nr-BLAST	nt-BLAST^a^	
F1	38,727	28,834	461	9,432
F2	30,684	22,641	459	7,584
F3	31,762	24,647	374	6,741
F4	40,378	27,934	549	11,895
F5	30,287	22,471	481	7,335
F6	32,855	23,436	491	8,928
F7	38,244	26,524	698	11,022

^a^Isotigs not matched to the NCBI nonredundant (nr) protein database

^b^No hits Indicate absence of homology to known sequences

**Fig. 4:**
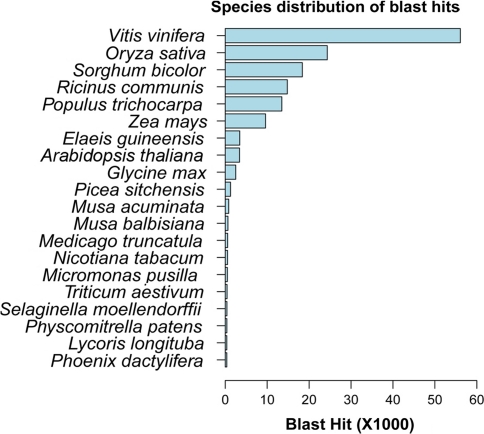
List of plant species whose cDNA sequence annotations contribute to our date palm isotig annotation effort. BlastX-based “top-hit” species are ranked by their matched entries. The highest “hit-species” is the grapevine, and only 0.2% of the Blast-hit match the date palm protein sequences due to a very limited data collection effort

For a further analysis on the BlastX results of the matching sequences, we found that our isotigs have the best matched to grapevine (*Vitis vinifera*) sequences, followed by rice (*Oryza sativa*) and Sorghum (*Sorghum bicolor*). But the latter two species only contributed less than half of the annotations; Grapevine-matched sequences account for 31.1% of annotated isotigs, while rice and sorghum only contribute 13.5 and 10.2% to the annotation, respectively. Although the oil palm (*E. guineensis*) sequences are expected to be most similar to those of date palm, only 1.9% of annotated isotigs were matched to them, which is mainly due to the limited amount of data available at the GenBank database (Fig. 4).

### Functional annotations of isotigs

**Fig. 5:**
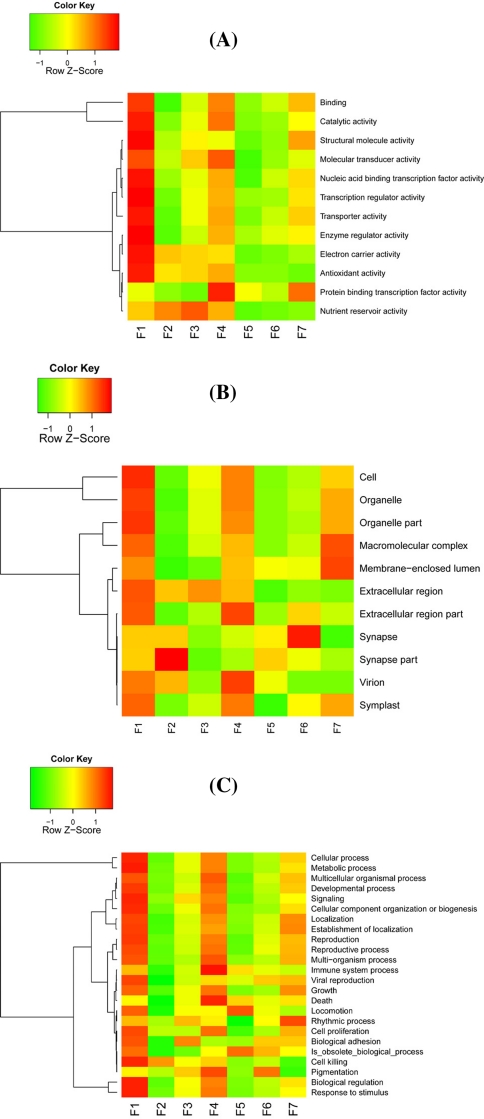
Expression profilles (heat-maps) of GO-based functional annotation (**a**, molecular function; **b**, cellular component; **c**, biological process). The color in the heatmap respresents Z-score normalized isotig number of each GO category. *Red* indicates high gene expression activitity, *yellow* indicates medium activity and *green* indicates low activity. GO categories were labled in the *left* of each heatmap line

We used the GO Consortium database for our functional annotations (http://www.geneontology.org), starting from the three root nodes: cellular component, biological process, and molecular function. First, taking molecular function as an example, we listed its 12 sub-categories (Fig. 5a). Among the seven stages, it is obvious that F1 is most transcriptionally active and the “binding” category alone involves 14,071 isotigs, which has 4,085 more isotigs than those of the F2 stage (9,986 isotigs; Additional file 3, Table S1). At the F1 stage, there are nine categories of genes that are expressed higher than those of other fruiting stages: binding, catalytic activity, structural molecule activity, nucleic acid binding transcription factor activity, transcription regulator activity, transporter activity, enzyme regulator activity, electron carrier activity and antioxidant activity. We observed several categories are expressed relatively high in some stages: nutrient reservoir activity of F3, molecular transducer activity and protein binding transcription factor activity of F4. Second, in the cellular component categories, there are more than 11,000 isotigs mapped to “cell” in each developmental stage, including the highest in F1 (16,053 isotigs), followed by F4 (15,098 isotigs), and the least in stage F2 (11,828 isotigs; Additional file 3, Table S1). When examining expression levels, we noticed that stages F1 and F4 have most highly expressed genes (Fig. 5b). We also can see that genes in categories synapse part are highly expressed in F2, genes in categories synapse are most expression in F6, and genes involved in membrane-enclosed lumen and macromolecular complex are relatively highly expressed in F7. Third, we investigated isotigs in cellular process that is the largest class of biological process, where again F1 expresses the highest number of genes (14,790 isotigs; Additional file 3, Table S1), followed by F4 (13,774 isotigs), and the least is F2 (10,717 isotigs). The second largest category is metabolic process, where the expression levels are ranked: F1 (14,334 isotigs), F4 (13,244 isotigs), and F7 (12,290 isotigs). To summarize, it is obvious that F1 is the most biologically active developmental stage (molecule biological process, cellular component, and biological process), followed by F4 and F7.

### Fruiting-associated genes

**Fig. 6:**
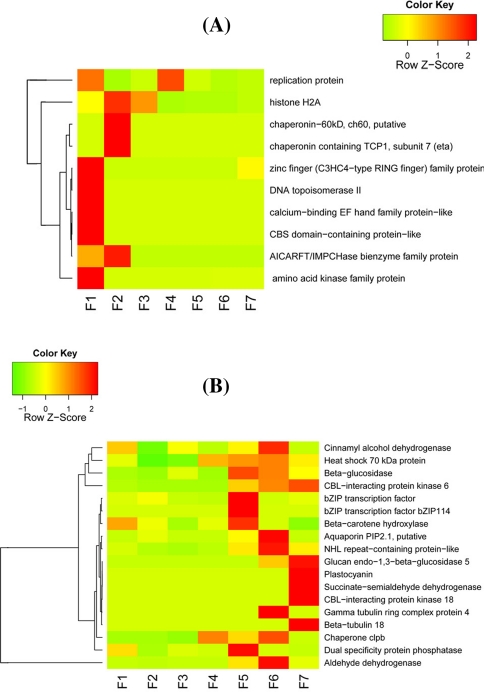
Expression profilles (heatmaps) of fruiting-associated genes (**a**, core cell cycle genes; and **b**, candidate fruit development-associated genes). The color in the heatmap respresents Z-score normalized isotig number. *Red* indicates high gene expression activitity, *yellow* indicates medium activity and *green* indicates low activity. Furuiting-related genes clusters were labled in the *left* of each heatmap line

**Fig. 7:**
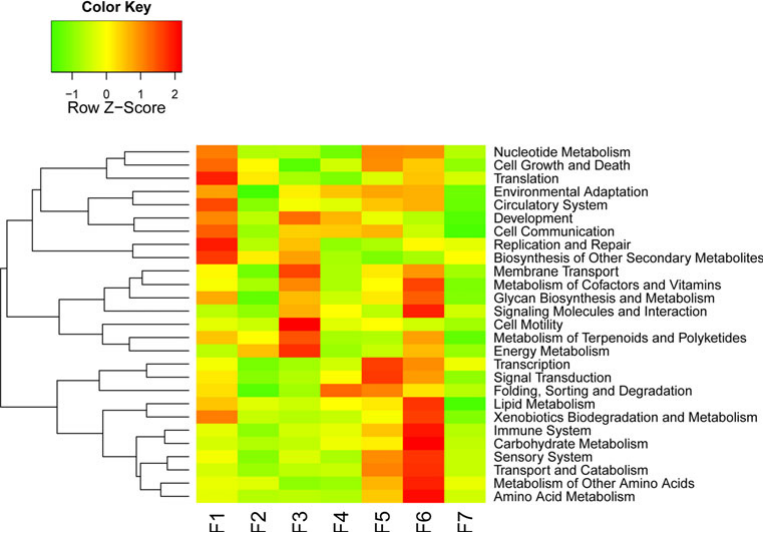
Clustering analysis (heatmap) of annotations for metabolic pathways (2nd level). The color in the heatmap respresents Z-score normalized isotig number of each KEGG pathway. *Red* indicates high gene expression activitity, *yellow* indicates medium activity and *green* indicates low activity. Furuiting-related genes clusters were labled in the *left* of each KEGG annotated pathway

**Fig. 8:**
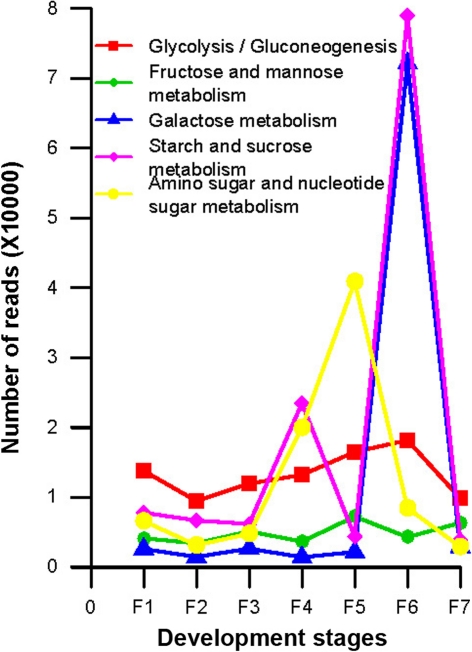
Sugar metabolism from carbohydrate metabolism (3rd level). Read coverage of each KEGG category at 3rd level, which is related to sugar metabolism

**Fig. 9:**
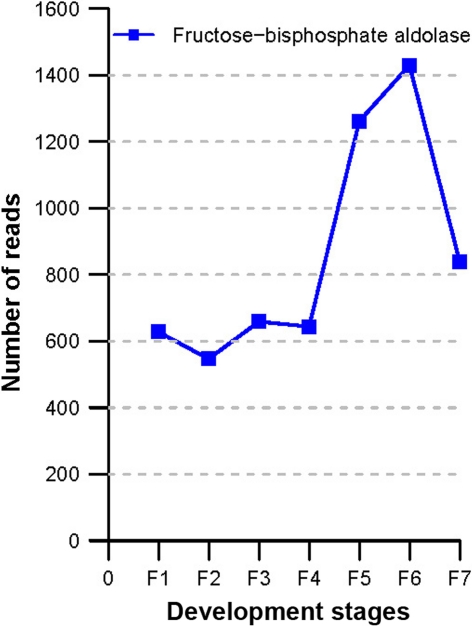
Fructose-bisphosphate aldolase expression at different fruiting stages

Since our goal here is not to do a comprehensive annotation for all genes, we selected a few classes of genes for further discussions. First, based on our BlastX results, we identified 10 core cell cycle-related genes and tried to see how they vary over the fruiting stages (Fig. 6a). It became obvious that stages F1 to F4 separate from F5 to F7, where the genes of the former group are very active but those of the latter group are quite opposite. What interesting is that fact the single zinc finger family protein is more relatively active than any other genes at the last fruiting stage. Second, we decided to interrogate 18 fruit ripening-associated genes (Fig. 6b). Expression of most of these genes gradually increased with the growing of the fruit, and reaching their peaks in the stage F5, F6, or F7; some of them are rather stage-specific and others are very informative, such as the zinc finger proteins at F5. Third, we also looked into KEGG metabolic pathways for secondary information clustering (Fig. 7). We observed that the F1 stage shows some highly expressed genes involved in number of important pathways such as replication/repair, translation, and cell growth/death. F6 is another stage that shows many metabolic activities, such as carbohydrate, amino acid, lipid, and cofactors/vitamins metabolisms. Fourth, we also stepped further to inspect sugar metabolism and found that genes involved in fructose/mannose, amino/nucleotide sugar metabolisms are expressed at highest level in stage F5, followed by genes in starch/sucrose/galactose metabolisms and glycolysis/gluconeogenesis, which peak at stage F6 (Fig. 8). Fifth, we list seven starch metabolism-related genes (Table 3), and the expression levels of these genes change differently during the fruit maturation process. At stage F1, starch synthesis appears strong as sucrose synthase, starch synthase, and sucrose phosphatase are all expressed at high levels, which are subsequently lowered, especially sucrose phosphatase detected only at stage F1. Sucrose-phosphate synthase, chloroplast beta-amylase, and alpha-glucosidase are expressed low initially but gradually increased when fruit ripens at stage F7. The UDP-glucose pyrophosphorylase gene expression is relatively flat to start and gradually increased to reach its peak at stages F4, F5, and F6, followed by a significantly reduction at stage F7. Sixth, fructose-bisphosphate aldolase is a very important enzyme in glycolysis (St-Jean et al. 2009). As showing in Fig. 9, it increases at F5, peaks at F6, and reduces significantly at F7. F6 is a critical period for the fruit to begin maturing and this gene reaches its peak at this time.

**Table 3:** Enzymes involved in starch metabolism

Enzyme	Expression level						
	F1	F2	F3	F4	F5	F6	F7
Sucrose synthase	1,822	1,060	306	900	39	84	92
Starch synthase	1,354	342	98	164	27	59	23
Sucrose phosphatase	124	0	0	0	0	0	0
UDP-glucose- pyrophosphorylase	204	169	254	396	484	494	273
Sucrose-phosphate synthase	2	7	0	14	728	141	34
Alpha-glucosidase	4	0	0	3	3	49	42
Chloroplast beta-amylase	93	39	70	290	299	1,232	301

### Experimental validation of selected differentially expressed genes

In order to validate in principle the gene expression profiling and developmental stage-associated genes identified based on sequencing alone (Roche/454 GS FLX), we selected several genes, and performed real-time PCR experiment. We chose 8 candidate genes for the validation: cell division control protein 2, cyclin-dependent kinase A, gamma-2 tubulin, 24-kDa seed maturation protein, fructose-bisphosphate aldolase, lipid transfer protein, starch synthase, and sucrose synthase (Additional file 4, Table S2). Overall, 5 genes from 8 showed a similar stage-associated trend, as what were observed in sequence analysis albeit not identical at all development stages (Additional file 5, Figure S3). For instance, the expression of fructose-bisphosphate aldolase at F7 is higher than F6, whereas it is the reverse in sequencing (additional file 4, Table S2). We believe that such variations are largely due to under-sampling of our sequencing approach and the more precise profiling has to be done with other short-tag and high-coverage platforms, such as those of the Illumina and Life Technologies. We noticed that the other 3 genes that failed to be validated (cell division control protein 2, cyclin dependent kinase A, and gamma-2 tubulin) are all expressed at a relatively lower level than those validated, and we have also known that real-time PCR validation is not sensitive enough for quantitate lowly expressed genes (An et al. 2011).

## Discussion

Aside from the high quality data we acquired, we are very grateful to the grape research community (Jaillon et al. 2007) whose series of studies since 2000 have provided invaluable data for gene annotation and functional evaluations, especially those transcriptome researches for fruit ripening (Ablett et al. 2000; Peng et al. 2007; da Silva et al. 2005; Iandolino et al. 2008).

For the GO functional classification, in molecular function category both of binding and catalytic activity of genes were expressed at highest level in F1 stage; in the cellular component category, cell was expressed at the highest level in F1 stage; in biological process category, both cellular process and metabolic process expressed at the highest level in F1stage (Additional file3, Table S1). The above information which indicates that cells are active for cell division in the F1 stage consistent with other results on apple (Janssen et al. 2008). In addition, the nutrient reservoir activity was higher in the first four stages of date palm fruit development, then the rest three developmental stages were significantly lower; the gene expression of channel regulator activity is higher in the first two developmental stages than the rest five developmental stages, which indicating that the fruit in the first few stages of development are devoted mainly for the energy reserve in preparation for the fruit development (Giovannoni 2004). The translation regulator activity only detected in the last three developmental stages, and these genes may be associated with the regulation of fruit ripening.

According to the annotation results, we identified 10 core cell cycle genes and 18 fruit ripening genes (Fig. 6). These core cell cycle genes expressed at a very high level in stage F1 and F2, then reduced in the subsequent developmental stages. Some investigators observed that after pollination, the cell will go through several rounds of rapid division, making a sharp increase in cells number (DENNE 1960, 1963). The regulation of cell cycle gene expression is complex, and it is possible that the core cell cycle gene transcript is involved in controlling cell division during fruit development (Menges et al. 2003, 2005; Menges and Murray 2002; Sorrell et al. 2001). Fruit ripening-related genes mainly expressed in stage F5, F6, and F7 (Fig. 6), including Beta-carotene hydroxylase and plastocyanin genes which are upregulated in the maturation process, indicating that these gene products play an important role in the fruit ripening process and the composition of the color of the fruit (Ampomah-Dwamena et al. 2009). Some investigators found that the pigments content (chlorophyll a + b and carotene) being high in early stages of development then greatly reduced in the late stages of development (Bacha et al. 1987). Surprisingly, their observation not matched with our result. This discrepancy indicates that carotene pigment is controlled by a number of genes and ß-carotene is just one of them. However, we noticed that some fruit ripening genes including ß-carotene, were expressed in F1 stage, and then nothing detactable until F5 stage and again disappeared in the late stages of development, suggesting that either the fruit development process is interrupted, or the products of these enzymes are further processed to generate other substances (Janssen et al. 2008).

In this study, we investigated seven starch metabolism-related genes (Table 3). From these results, we found that sucrose synthase, starch synthase, and sucrose phosphatase are expressed at a high level in F1 stage, then in subsequent stages, sucrose synthase and starch synthase reduced, while sucrose phosphatase was completely undetectable indicating that large amounts of starch is accumulated at this stage to prepare and ensure the smooth development of the fruit. The expression level of other four genes is peaked at stage F5 or F6, which is close to maturity, indicating that these genes regulate the starch metabolism during the fruit development. Even, many of amylase biochemical activities have been described before (Purgatto et al. 2001; Peroni et al. 2008; Wegrzyn et al. 2000); it is difficult to determine how those enzymes regulate the starch level during fruit development.

Metabolic pathway analysis (KEGG) is a reference base functional annotation linked to the interaction network of biological systems (Kanehisa et al. 2006; Moriya et al. 2007). We found that some metabolic pathways such as replication and repair, translation, and cell growth and death, etc. are mostly active in F1 stage, then decreased in the next developmental stage (Fig. 7), indicating that there are rapid cell divisions during this developmental stage. Cell motility, membrane transport, and development are most active in F3 stage; also in this stage the fruit has a rapid growth in its appearance. The pathway of folding, sorting and degradation is the most active at F4 stage, which suggests that many biological reaction, such as chaperones, folding catalysts, protein export and RNA degradation and so on, occurred at this stage of fruit ripening. Stage F5 and F6 are critical periods for the fruit ripening, and the genes in many pathways showed a very high activities, especially the pathways related to the nutrients accumulation, including sugar. In addition, biosynthesis of other secondary metabolites was very active in stage F6 e.g. immune system, transport and catabolism and others (Fig. 7).

## Methods

### Plant materials

The date palm fruits of Khalas at seven different developmental stages were collected from Al-Hssa Oasis (25°04′35″N, 49°06′24″E) and Al-Kharj (24°08′54″N, 47°18′18″E), Saudi Arabia. After thorough washing with distilled water, the samples were immediately frozen in liquid nitrogen and transported to the laboratory. The samples were stored at −80°C until use.

### RNA isolation and cDNA synthesis

We used a standard CTAB (2%) method to isolate total RNAs. After the removal of DNA with DNaseI treatment, we quantified the RNA using a NanoDrop™ Spectrophotometer ND-8000, and followed by checking on 1% agarose gel. mRNA was isolated from the total RNA by using an Oligotex mRNA mini kit (Qiagen). To reduce the content of rRNA (Cloonan et al. 2008), we treat the mRNA using RiboMinusTM Plant Kit (Invitrogen), and the samples were checked again by using Agilent 2100 bioanalyzer with Agilent RNA 6000 Nano Kit (Agilent).

### cDNA library construction and sequencing

We used at least 200 ng of high quality mRNA for each library construction. The mRNA was fragmented by incubating at 70°C in fragmentation buffer to yield a size range of 450–1,200 bp, checked by using Agilent RNA 6000 Pico Chip (Agilent). We used Rapid cDNA library kits (Roche) for the library construction. The cDNA library was heated at 95°C for 2 min and chilled on ice immediately before the emPCR procedure. Sequencing was performed on a Roche/454 Genome Sequencer FLX Titanium Instrument following its standard protocols (Margulies et al. 2005). Files containing sequence reads and quality scores were deposited in the Short Read Archive of the National Center for Biotechnology Information (NCBI) [Accession number SRA045434.3].

### Sequence assembly and data processing

We assembled all sequencing reads using the Roche/454 Newbler version 2.5 (Kumar and Blaxter 2010). The parameters we used were “-cdna –ace –urt -ud –tr”, and the isotig files generated by the Newbler assembler were processed further. We compared Isotigs with the protein non-redundant database (NCBI; Bethesda, MD, USA) by using BlastX (Altschul et al. 1997) (E value ≤ 1e − 5) for annotation. The unmatched isotigs or unigenes were searched against the nt database by using BlastN (Altschul et al. 1990) with a cutoff of 1E − 10. We annotated metabolic pathways using Kyoto Encyclopedia of Genes and Genomes (http://www.genome.jp/kaas-bin/kaas_main?mode=est_b) (Moriya et al. 2007). Gene ontology (GO) terms (Ashburner et al. 2000) were extracted from the best hits obtained from the BlastX against the uniprot database (E value ≤ 1e − 5), which were then sorted for different GO categories. The accumulative isotig number of each KEGG and GO category was collected. The categories that have less than 10 isotigs in all seven stages were first filtered to reduce background noises, and then the remaining isotigs were normalized using the Z-score method with the default parameters in R. Hierarchical clustering of genes in each KEGG or GO category was used to generate dendrograms based on Z-score using the heatmap.2 package and the default complete linkage method in R.

### Real time PCR

Primers for the 8 important candidate genes were designed using Oligo6 software based on corresponding sequences we generated in this study. The cDNA from F1 to F7 samples are all same with that used in the transcriptome sequencing experiment. Real-time PCR were performed at an annealing temperature of 60°C and 40 amplification cycles in 3 duplicates by using SYBR Green master mix (AB, Life technology) with 7900HT fast real-time PCR system (AB, Life technology).

## Electronic supplementary material

Below is the link to the electronic supplementary material.

Supplementary material 1 (PDF 21 kb) An overview of the data. (A) Reads length distribution; (B) Reads of GC content; (C) Isotigs length distribution; (D) Isotigs GC content

Supplementary material 2 (PDF 15 kb) Data saturation curve. From 50,000 to the total number of GS FLX reads are used for assembly by using Newbler 2.5

Supplementary material 3 (PDF 1948 kb) Real-time PCR validation of 8 selected genes. X axis is fruit development stages from F1 to F7, Y axis is relative expression detected by real-time PCR. (A) Cell division control protein 2; (B) Cyclin dependent kinase A; (C) Fructose-bisphosphate aldolase, (D) lipid transfer protein; (E) 24 kDa seed maturation protein; (F) starch synthase; (G) sucrose synthase; (H) Gamma-2 tubulin

Supplementary material 4 (XLSX 13 kb)

Supplementary material 5 (XLSX 10 kb)
