# Supplementary figures and images for: High-throughput sequencing-based gene profiling on multi-staged fruit development of date palm (Phoenix dactylifera, L.)
Source: Plant Mol Biol. 2012 Feb 21;78(6):617–26. doi: 10.1007/s11103-012-9890-5 (PMC3313043; doi:10.1007/s11103-012-9890-5)

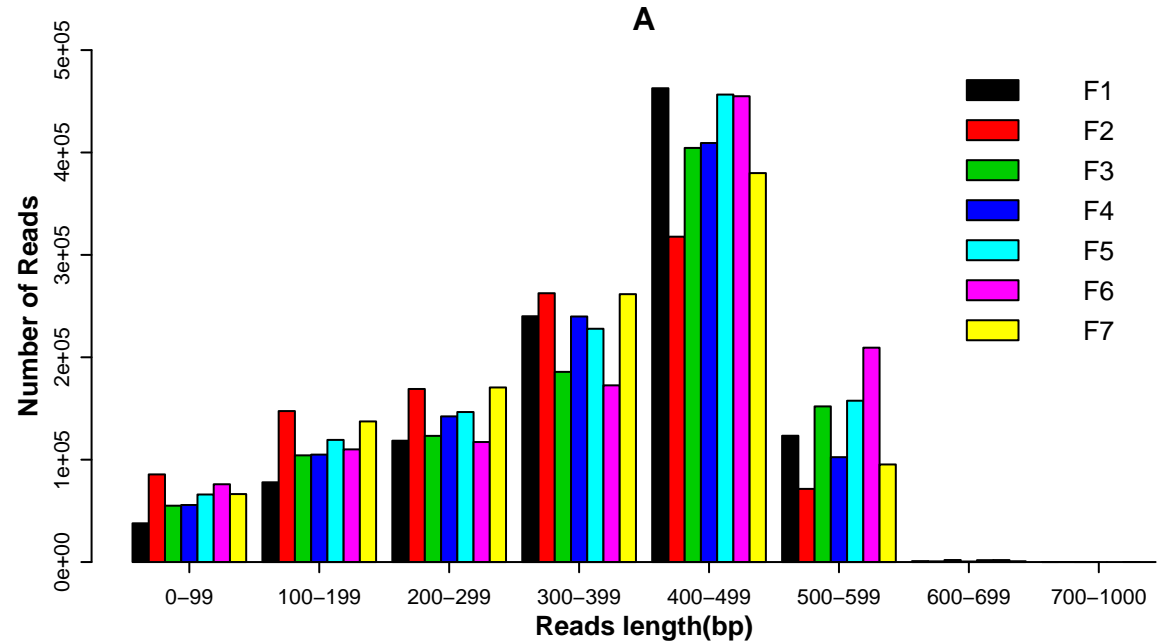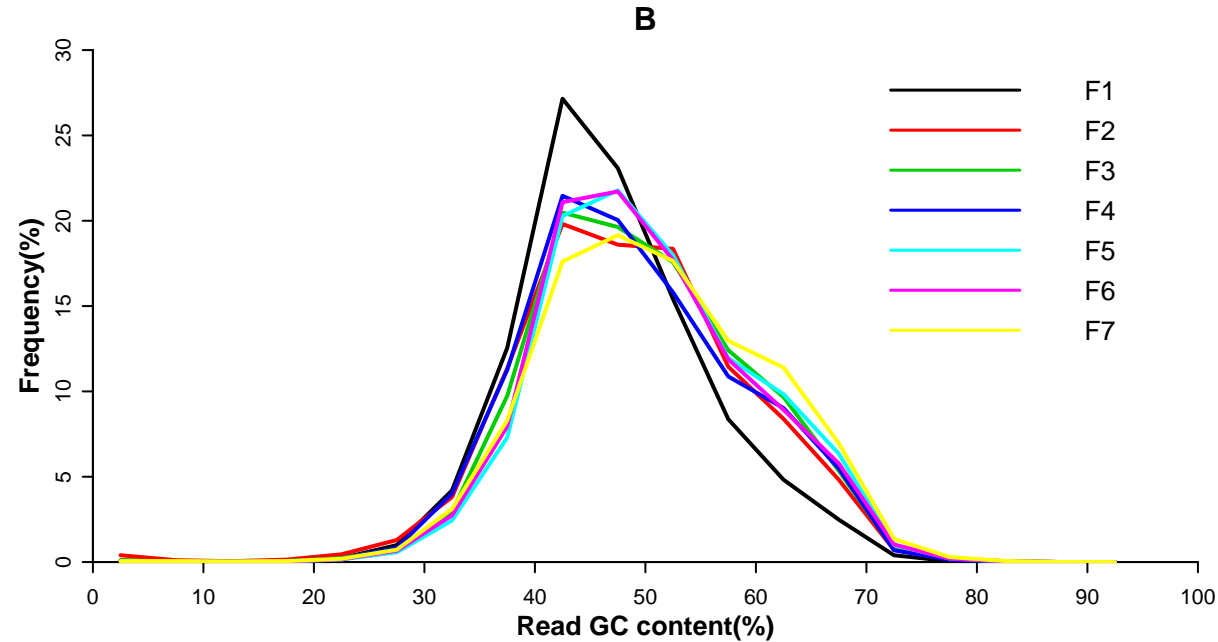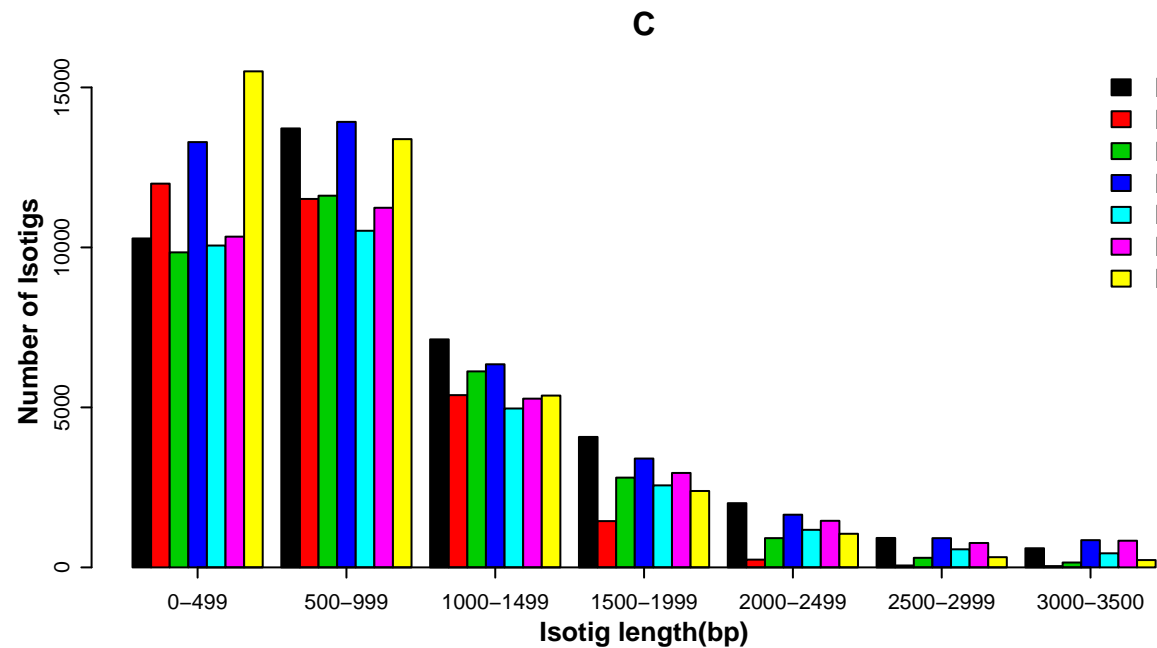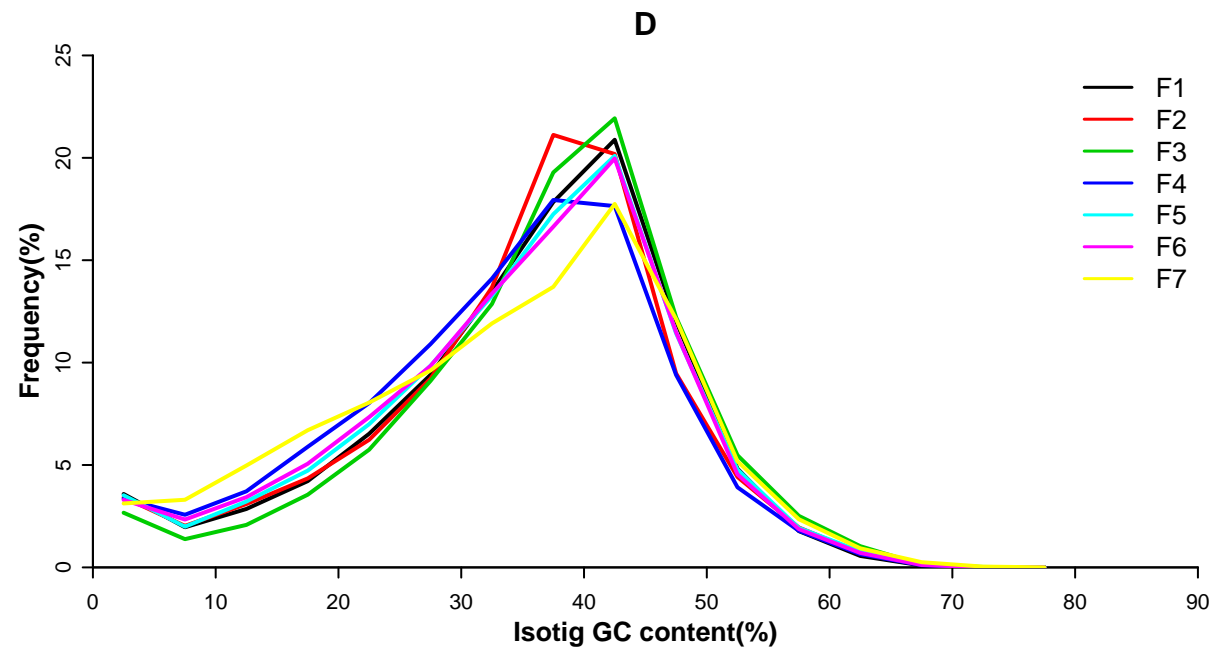

Supplement: Supplementary file 1 — Supplementary material 1 (PDF 21 kb) An overview of the data. (A) Reads length distribution; (B) Reads of GC content; (C) Isotigs length distribution; (D) Isotigs GC content [file 11103_2012_9890_MOESM1_ESM.pdf]

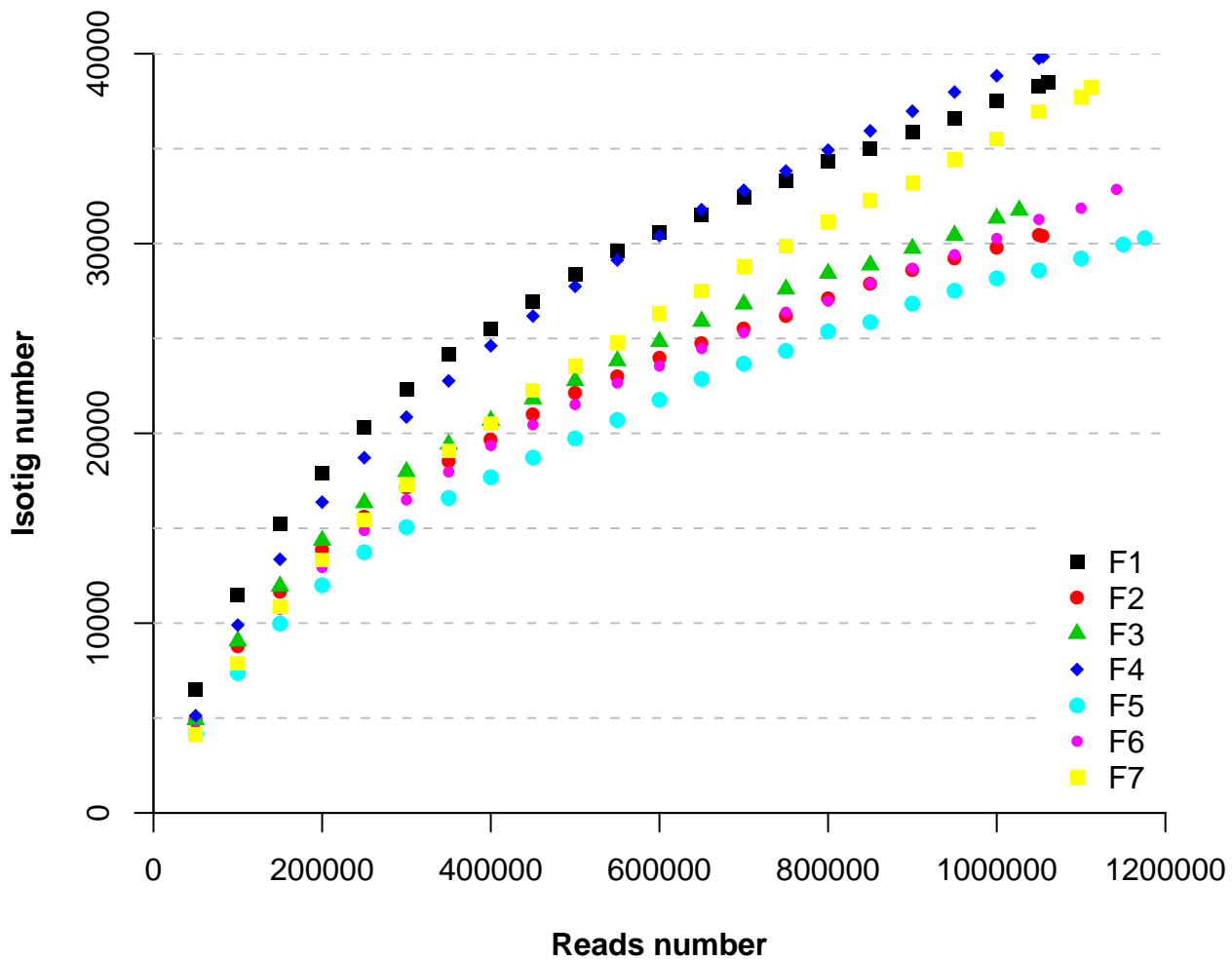

Supplement: Supplementary file 2 — Supplementary material 2 (PDF 15 kb) Data saturation curve. From 50,000 to the total number of GS FLX reads are used for assembly by using Newbler 2.5 [file 11103_2012_9890_MOESM2_ESM.pdf]

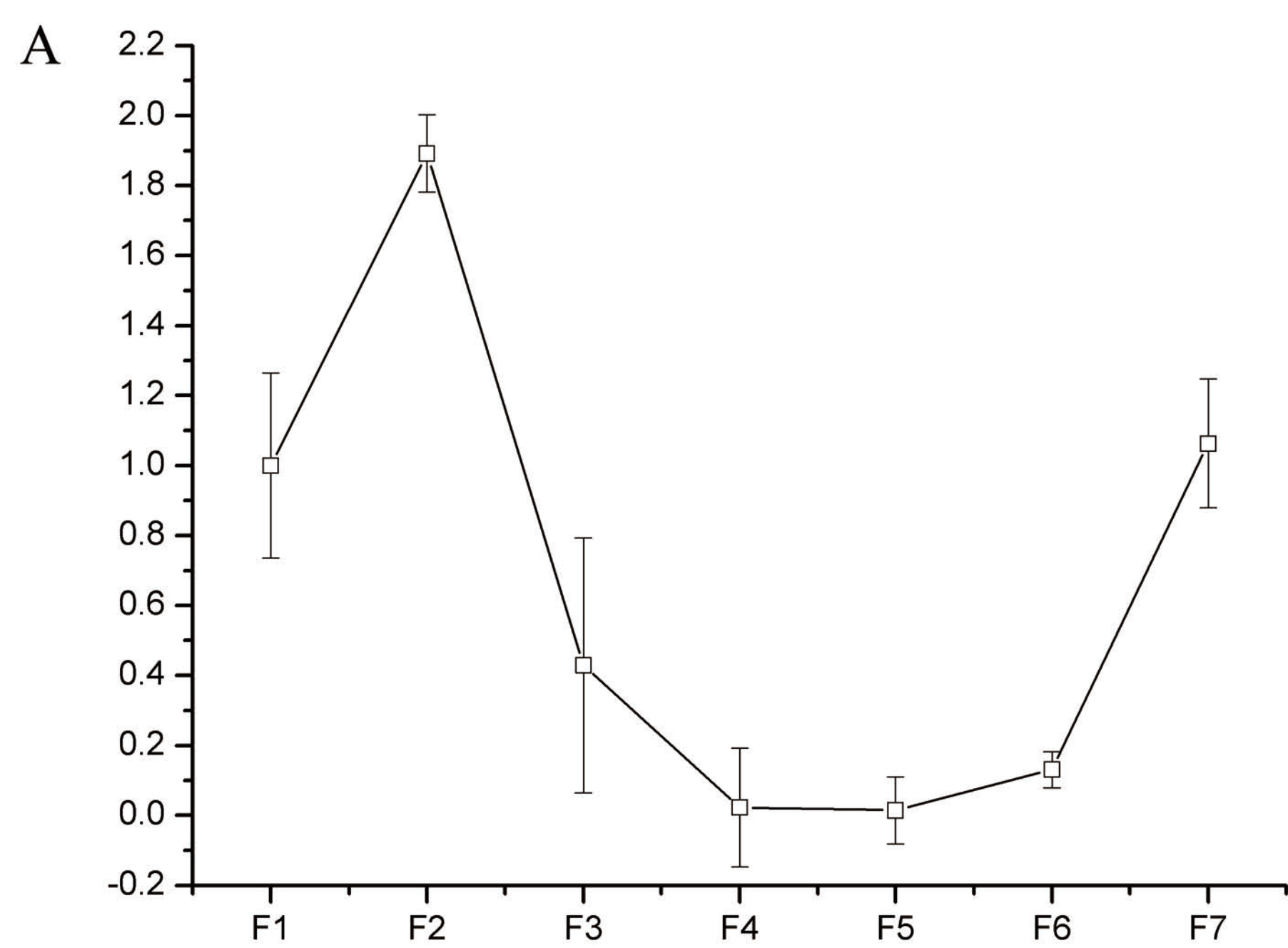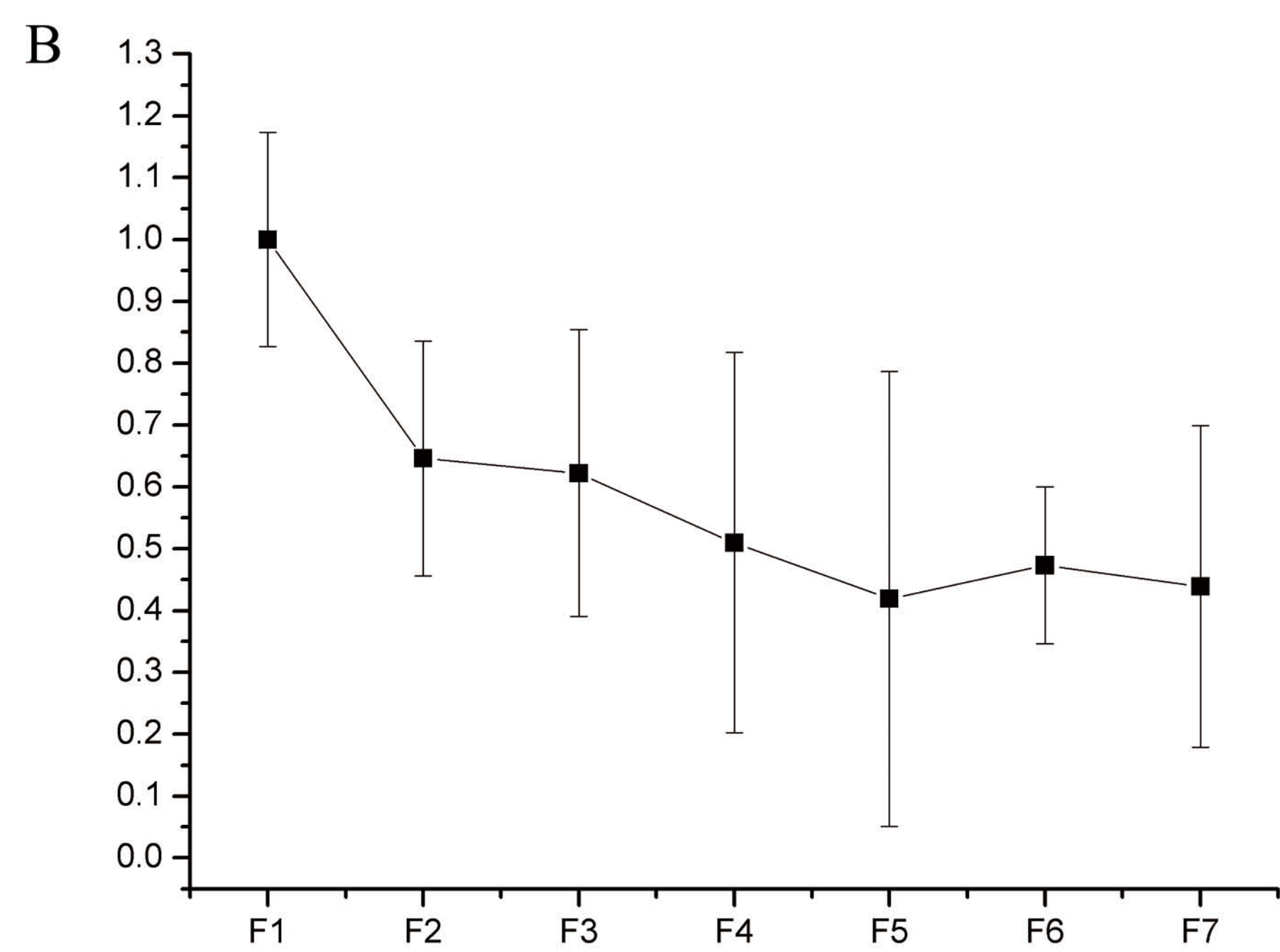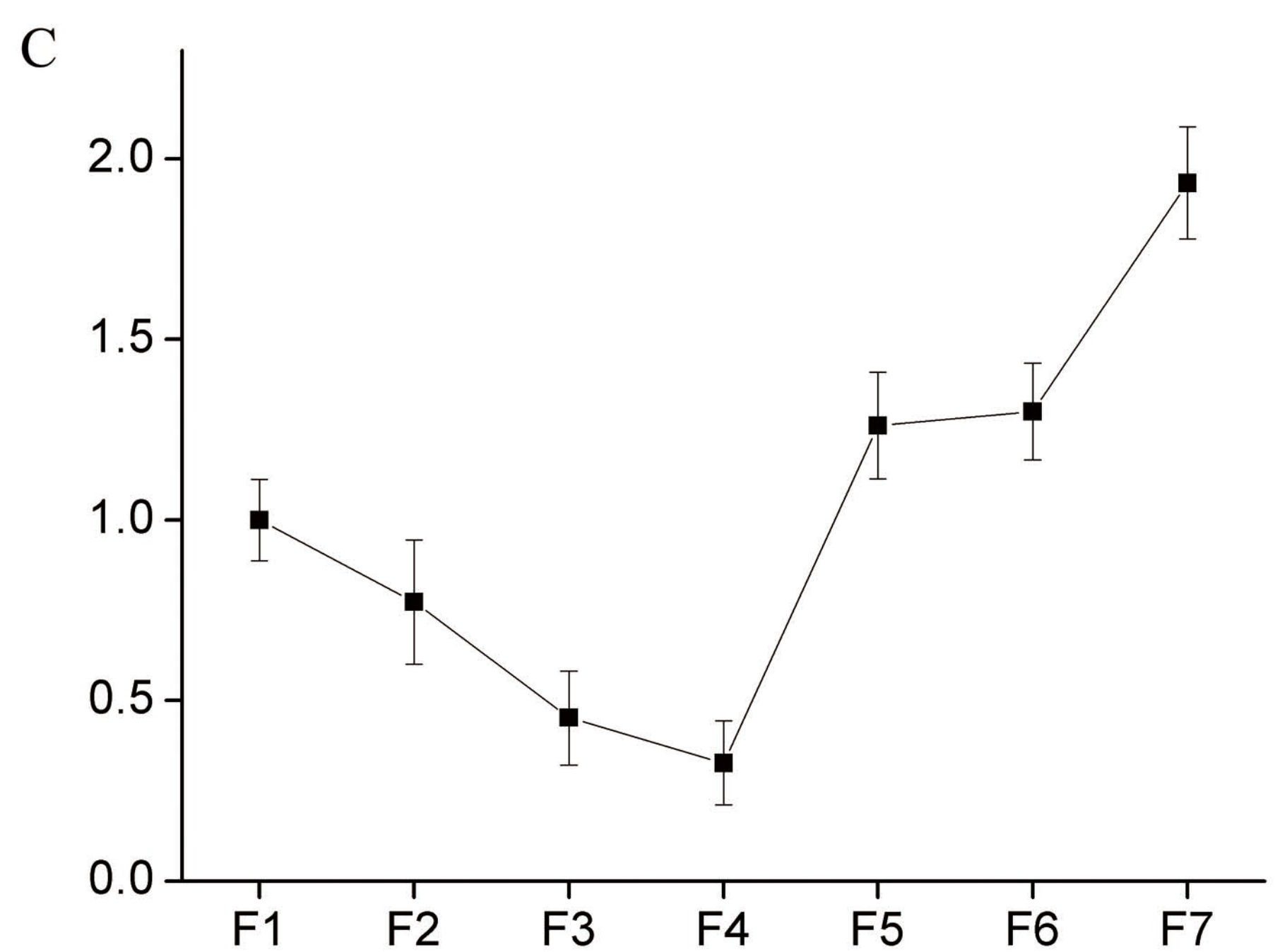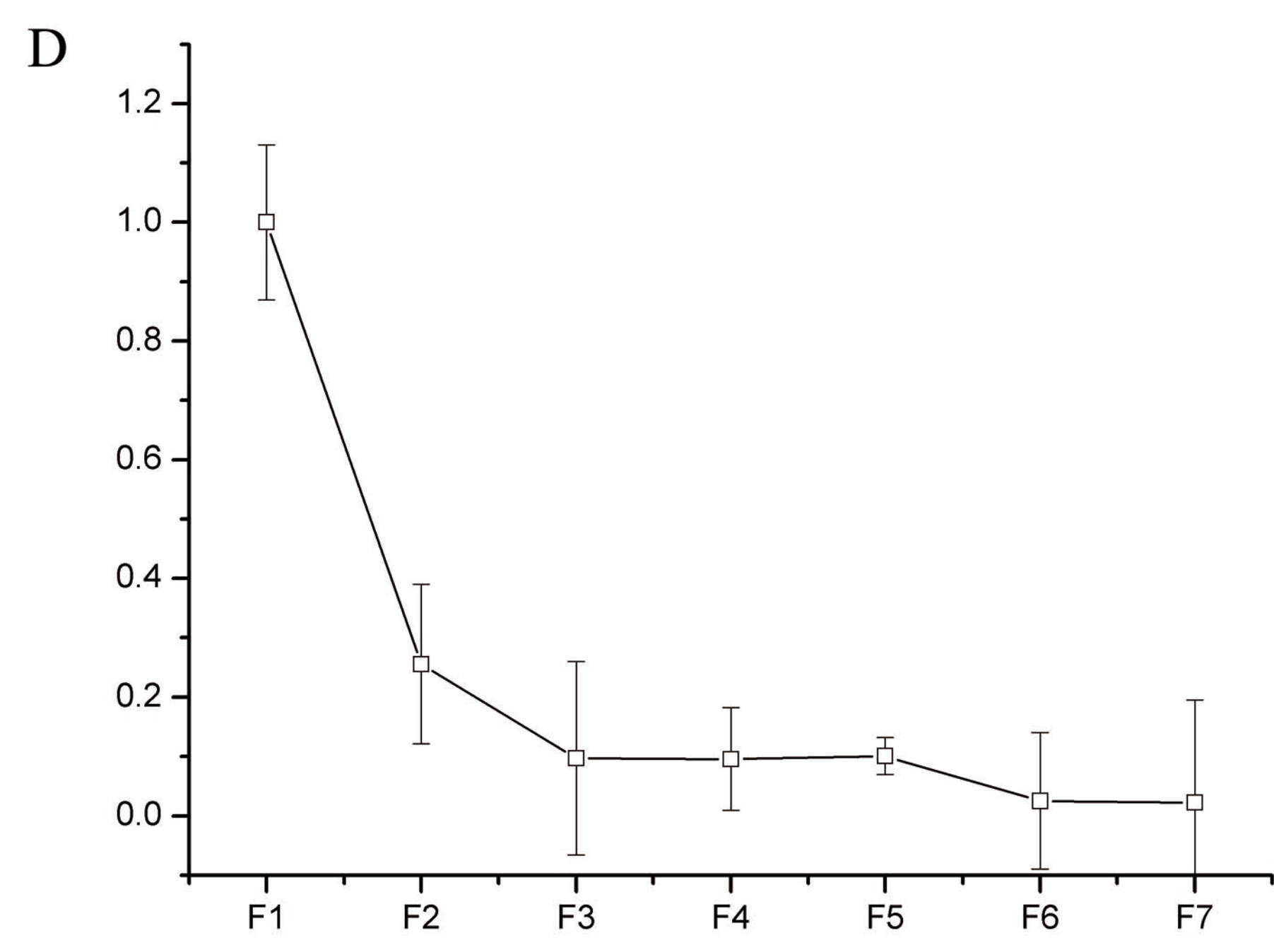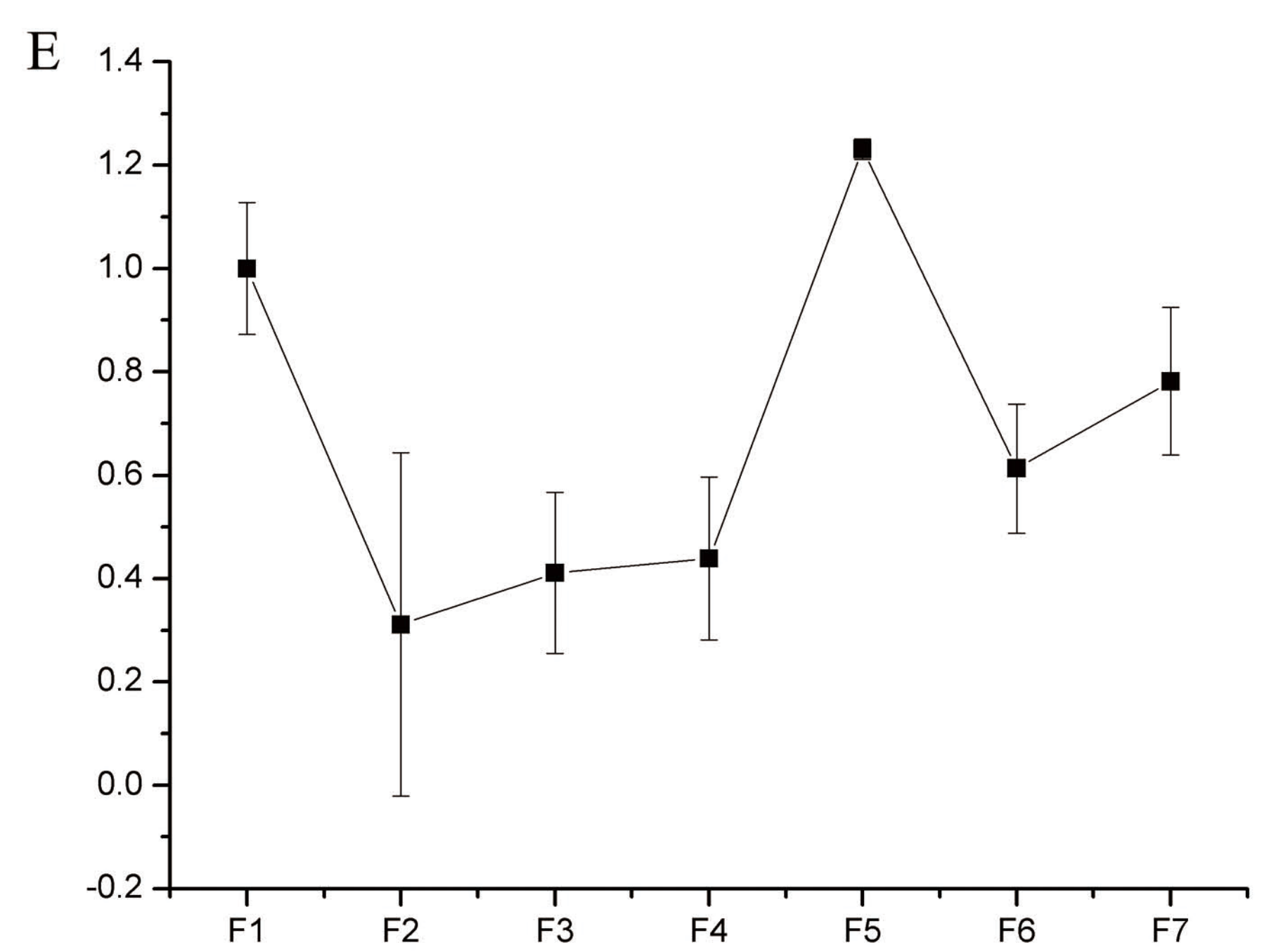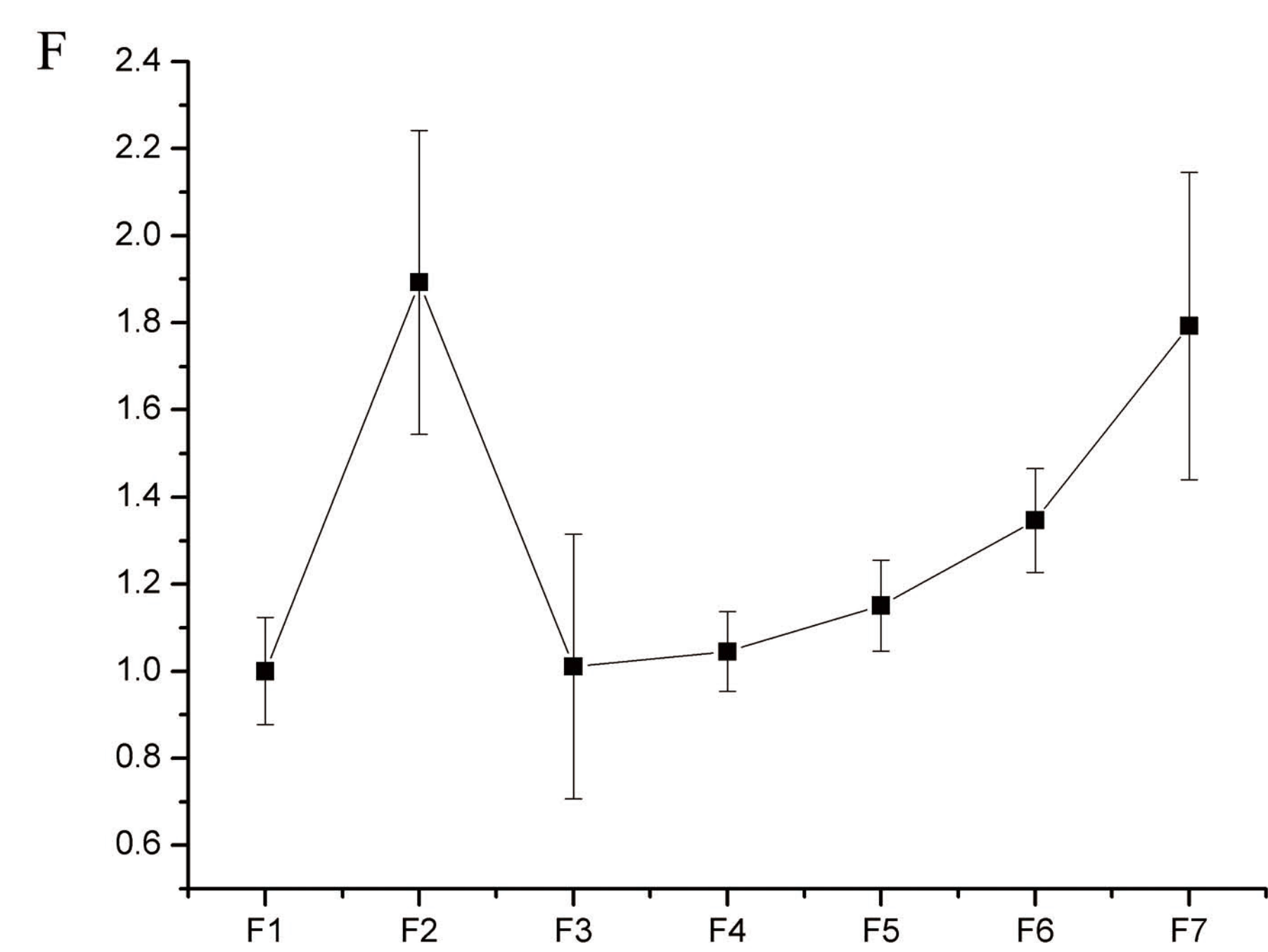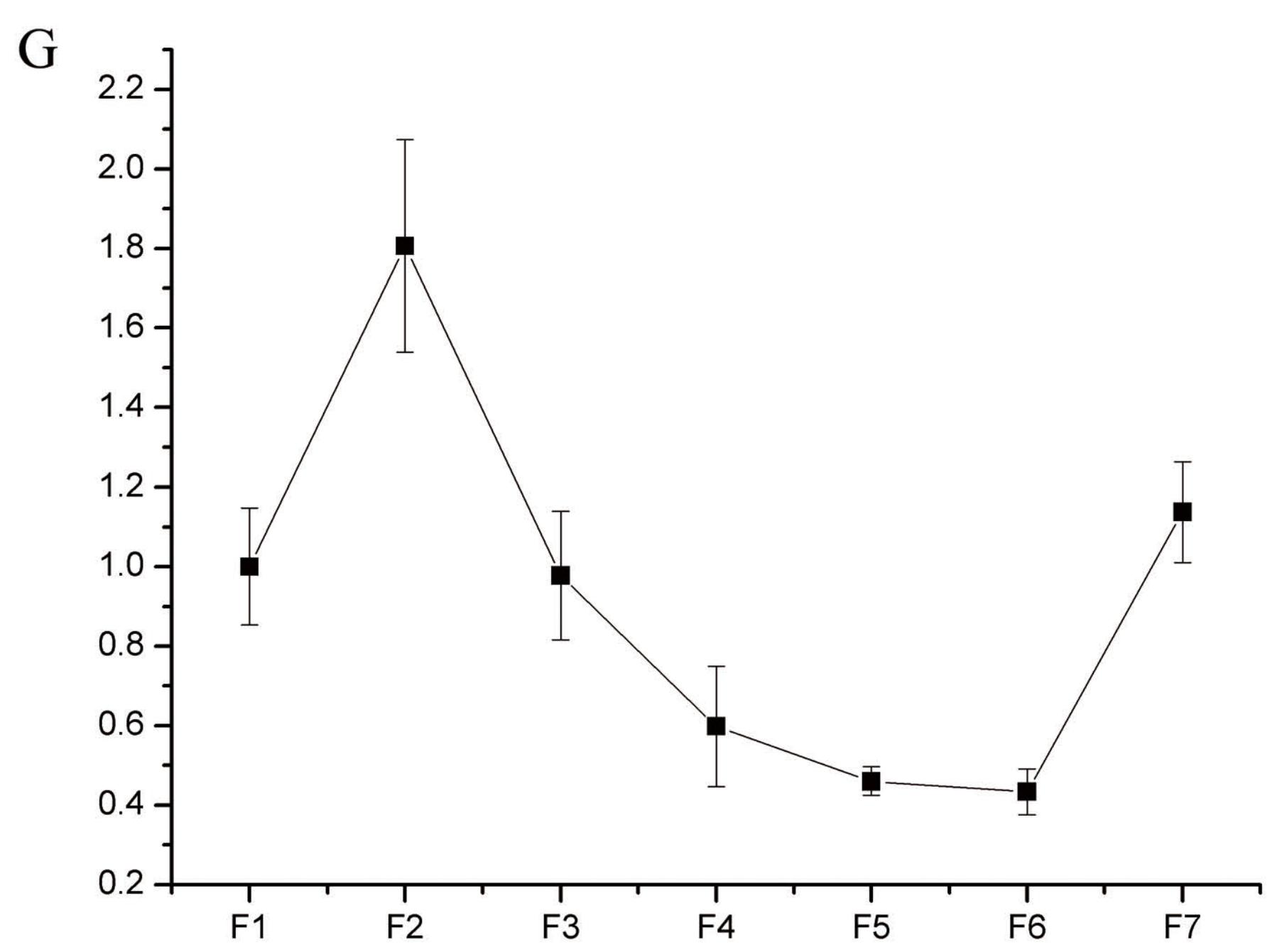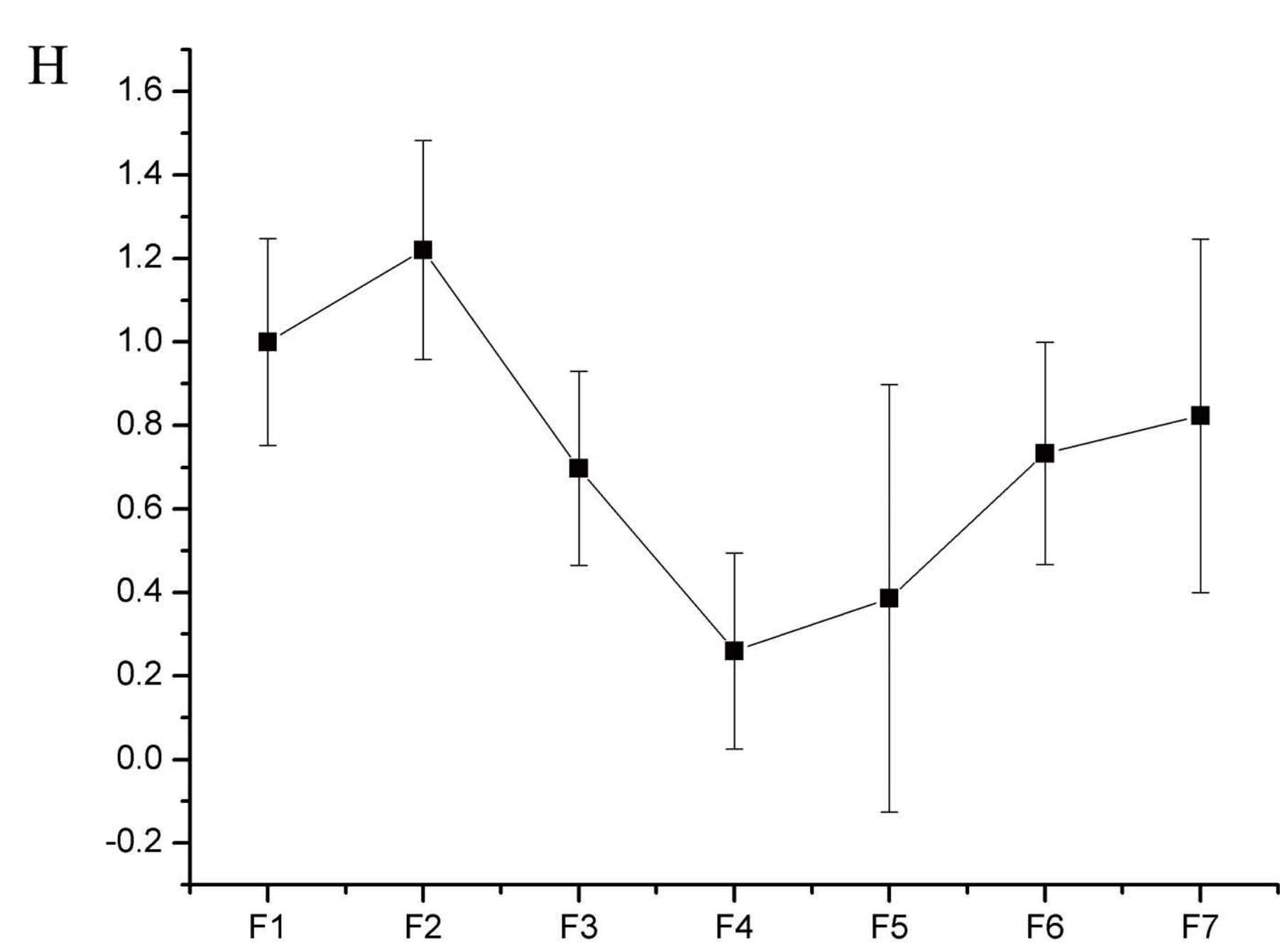

Supplement: Supplementary file 3 — Supplementary material 3 (PDF 1948 kb) Real-time PCR validation of 8 selected genes. X axis is fruit development stages from F1 to F7, Y axis is relative expression detected by real-time PCR. (A) Cell division control protein 2; (B) Cyclin dependent kinase A; (C) Fructose-bisphosphate aldolase, (D) lipid transfer protein; (E) 24 kDa seed maturation protein; (F) starch synthase; (G) sucrose synthase; (H) Gamma-2 tubulin [file 11103_2012_9890_MOESM3_ESM.pdf]
